# Exercise training for asbestos-related and other dust-related respiratory diseases: a randomised controlled trial

**DOI:** 10.1186/1471-2466-14-180

**Published:** 2014-11-18

**Authors:** Marita T Dale, Zoe J McKeough, Phillip A Munoz, Peter Corte, Peter T P Bye, Jennifer A Alison

**Affiliations:** Clinical and Rehabilitation Sciences, Faculty of Health Sciences, The University of Sydney, Sydney, NSW Australia; Physiotherapy Department, St Vincent’s Hospital, Sydney, NSW Australia; Department of Respiratory Medicine, Royal Prince Alfred Hospital, Sydney, NSW Australia; Sydney Medical School, The University of Sydney, Sydney, NSW Australia; Department of Physiotherapy, Royal Prince Alfred Hospital, Sydney, NSW Australia

**Keywords:** Asbestosis, Diffuse pleural thickening, Interstitial lung disease, Exercise, Pulmonary rehabilitation, Physical activity, Health-related quality of life

## Abstract

**Background:**

The study aimed to determine the short and long-term effects of exercise training on exercise capacity and health-related quality of life (HRQoL) compared to usual care in people with dust-related pleural and interstitial respiratory diseases. No previous studies have specifically evaluated exercise training in this patient population.

**Methods:**

Participants with a diagnosis of a dust-related respiratory disease including asbestosis and asbestos related pleural disease were recruited and randomised to an eight-week exercise training group (EG) or a control group (CG) of usual care. Six-minute walk distance (6MWD), St George’s Respiratory Questionnaire (SGRQ) and Chronic Respiratory Disease Questionnaire (CRQ) were measured at baseline, eight weeks and 26 weeks by an assessor blinded to group allocation.

**Results:**

Thirty-three of 35 male participants completed the study. Sixty-nine percent of participants had asbestos related pleural disease. At eight weeks, compared to the CG, the EG showed a significantly increased 6MWD (mean difference (95%CI)) 53 metres (32 to 74), improved SGRQ total score, -7 points (-13 to -1) and increased CRQ total score, 6.4 points (2.1 to 10.7). At 26 weeks significant between-group differences were maintained in 6MWD, 45 metres (17 to 73) and CRQ total score, 13.1 points (5.2 to 20.9).

**Conclusion:**

Exercise training improved short and long-term exercise capacity and HRQoL in people with dust-related pleural and interstitial respiratory diseases.

**Clinical trial registration number:**

ANZCTR12608000147381. Date trial registered: 27.03.2008.

## Background

Dust-related pleural and interstitial respiratory diseases are a global health problem. Past and ongoing exposure to harmful dusts continue to impact millions of people worldwide [1–3] with the prevalence of some dust-related respiratory diseases increasing [4]. The pathological changes of these diseases include parenchymal and/or pleural fibrosis which may result in shortness of breath at rest or on exertion, reduced exercise capacity and health-related quality of life (HRQoL) [5–8]. Limited treatment options are available for people with dust-related pleural and interstitial respiratory diseases [3, 9].

Interstitial lung disease (ILD) describes a broad range of diseases with diverse aetiologies including idiopathic pulmonary fibrosis (IPF). Recently, exercise training has been shown to improve exercise capacity and HRQoL in people with ILD, predominantly IPF [10, 11]. While there is an indication from uncontrolled or mixed-disease studies that exercise training may benefit people with dust-related interstitial respiratory diseases [12, 13], no studies using rigorous randomised controlled trial methodology have investigated the effect of exercise training on exercise capacity and HRQoL in people with dust-related pleural and interstitial respiratory diseases.

Physical activity levels play an important role in health outcomes in people with chronic respiratory diseases. Higher levels of physical activity are associated with health benefits for people with chronic obstructive pulmonary disease (COPD), including greater exercise capacity and a higher single breath diffusing capacity for carbon monoxide (D_L_CO) [14]. Conversely, lower levels of physical activity are associated with increased morbidity and mortality [15]. The effect of exercise training on physical activity levels in people with dust-related pleural and interstitial respiratory diseases is unknown.

The primary aims of this study were to determine the short and long-term effects of exercise training compared to usual care on exercise capacity and HRQoL in people with dust-related pleural and interstitial respiratory diseases. The secondary aims were to determine whether exercise training improved symptoms and habitual daily physical activity compared to usual care in people with dust-related pleural and interstitial respiratory diseases.

## Methods

### Study design

The study was a multi-centred, assessor blinded, parallel group, randomised controlled trial and was conducted in New South Wales, Australia between January 2009 and July 2011. After enrolment into the study and collection of baseline measures, participants were randomly allocated to either an exercise group (EG) or control group (CG) by a person independent of the recruitment process through a computer-generated randomisation programme. Random allocation with minimisation for disease type (asbestos related pleural disease or other), severity (FVC <80% or ≥80%) and training centre was used.

The study was approved by the Human Research Ethics Committee of Sydney South West Area Health Service and all participants gave written informed consent. The trial was registered with the Australian and New Zealand Clinical Trials Registry (ANZCTR12608000147381).

### Participants

Participants were recruited through the Workers’ Compensation Dust Diseases Board (DDB) of New South Wales, Australia, respiratory physicians, support groups, workers’ unions and newsletters for returned servicemen. Both males and females were eligible to participate if they had a medical diagnosis of a non-malignant dust-related pleural or interstitial respiratory disease including asbestosis, silicosis and asbestos related pleural disease (defined as diffuse pleural thickening and/or rounded atelectasis). Diagnosis had been established by the participant’s respiratory physician or by the DDB Medical Authority which is a panel of respiratory physicians with specialist knowledge in occupational lung disease. The diagnostic process included an occupational history, clinical examination by a physician, radiological findings on chest X-ray and computed tomography scans confirming dust-related pleural and/or interstitial disease, and lung function (spirometry and lung volumes via plethysmography) as previously described [16].

People were excluded from the study if they had mesothelioma; discrete parietal pleural plaques as their only manifestation of dust exposure; cardiovascular, neurological or orthopaedic conditions limiting exercise performance; were on long term oxygen therapy; could not understand English; or had participated in a pulmonary rehabilitation programme within the last 12 months.

### Intervention

The EG participated in individually prescribed exercise training at one of seven pulmonary rehabilitation programmes. All participants completed supervised aerobic exercise training, including both walk and cycle training, three times weekly for eight weeks. The training mode, intensity and duration were based on the recommended guidelines for COPD [17]. For walking training the exercise intensity was 80% of the average walking speed of the better baseline six-minute walk test (6MWT) and for cycle training the initial intensity was 60% of peak work rate achieved on the incremental cycle test (ICT). Intensity was progressed weekly by increments of 5% of the average walking speed or the peak work achieved on the ICT, or if dyspnoea or rate of perceived exertion (RPE) scores (whichever was highest) was less than three on the modified Borg 0-10 scale [18] during an exercise training session. Participants completed a minimum of 15 minutes walking and 15 minutes cycling at each session in the first training week, progressing to 30 minutes of each modality by the final week. Rest periods were permitted as required. Adherence with training was pre-defined as completion of at least 80% of training sessions. Exercise training did not include strength training and there was no education component. At the completion of the eight week training the EG was not provided with any further advice or guidance on continuing exercise training. The CG did not receive any exercise intervention and continued with their usual medical management for the duration of the study.

### Pulmonary function

Participants performed spirometry and lung volumes (body plethysmography) at baseline and eight weeks and D_L_CO at baseline only (SensorMedics, Yorba Linda, Ca, USA). Spirometry was also measured at 26 weeks following the intervention period. All tests were performed according to standard protocols [19] and results expressed as a percentage of the predicted values [19–21].

### Outcome measures

All participants attended Royal Prince Alfred Hospital (RPAH), Sydney, Australia, for testing at baseline, immediately following eight weeks of exercise training or usual care and again after a further 26 weeks. All outcomes were measured by the same assessor, blinded to group allocation. The primary outcomes for the study were exercise capacity measured by the six-minute walk distance (6MWD) and HRQoL measured by the Chronic Respiratory Disease Questionnaire (CRQ) total score and the St George’s Respiratory Questionnaire (SGRQ) total score. Secondary outcomes were peak work rate achieved on the ICT, endurance cycle time, CRQ dyspnoea, fatigue, emotional function and mastery domain scores, SGRQ symptoms, activity and impact domain scores and physical activity measured by an activity monitor. Whilst all primary and secondary outcomes were determined at baseline and eight weeks following the interventions, only 6MWD, HRQoL and physical activity outcomes were measured after a further 26 weeks.

### Exercise capacity

Participants performed two six-minute walk tests (6MWT 1 and 6MWT 2) according to guidelines [22] on a continuous 32-metre oval track at baseline, immediately following the exercise training or eight weeks of usual care period and again after a further 26 weeks. Tests were separated by a minimum of 30 minutes or until heart rate and oxygen saturation had returned to resting values.

On a separate day, participants performed a symptom-limited ICT and endurance cycle test (ECT) on an electromagnetically-braked cycle ergometer (Lode BV, Groningen, The Netherlands). Tests were separated by a minimum of one hour or until heart rate and oxygen saturation had returned to resting values. For the ICT, work rate was increased every minute by a predetermined amount, ranging from 5 to 20 Watts.min^-1^ according to the participant’s self-reported exercise capacity and clinical judgement so that test duration was approximately 10 minutes [23]. For the ECT, work rate was increased to 40% peak work rate in the first minute and to 80% peak work rate from the second minute to symptom-limited test termination [24]. During the ICT and ECT participants wore a face mask and breath-by-breath measures for oxygen uptake (VO_2_) and carbon dioxide output (VCO_2_) were obtained (Vmax Encore, SensorMedics, Yorba Linda, USA). Heart rate and SpO_2_ were simultaneously measured using a pulse oximeter (Radical™, Masimo Corporation, Irvine, USA). Dyspnoea and RPE scores were recorded each minute and at peak work rate using the modified Borg 0-10 scale [18]. Peak work rate from the ICT was compared to predicted normal values [25]. The ICT and ECT were only performed at baseline and eight weeks.

### Health-related quality of life (HRQoL)

Participants completed the Chronic Respiratory Disease Questionnaire (CRQ) and the St George’s Respiratory Questionnaire (SGRQ) in random order at baseline, eight and 26 weeks. Both questionnaires have previously been used to evaluate HRQoL in ILD [7]. The minimal important difference (MID) for improvements in HRQoL in people with dust-related pleural and interstitial respiratory diseases has not been established. However, in people with COPD, a change of 0.5 in the mean score per item for the CRQ (calculated by dividing the overall score by the number of questions) has been established as the (MID) [26]. If the actual scores, rather than mean score, are used, the MIDs for each domain are 2.5 for dyspnoea, 2 for fatigue, 3.5 for emotional function, 2 for mastery, and 10 for the CRQ total score. For the SGRQ, the MID is a reduction of four points in the SGRQ total score for people with COPD.

### Physical activity

Physical activity was measured using an activity monitor (SenseWear Pro3 Armband, BodyMedia, Pittsburgh, PA, USA) worn for seven consecutive days when not attending for exercise testing at baseline, eight and 26 weeks. Participants were instructed to wear the activity monitor continuously, removing it only when showering or swimming. Steps per day, metabolic equivalents (METs) and energy expenditure were recorded using the SenseWear Professional software Version 6.1. Compliance was set at a minimum wear time of >85% per day for at least three days [27]. If this level of compliance was not achieved, the data were excluded from analysis.

### Statistical analysis

Data were analysed according to the intention-to-treat principle. Statistical analysis was performed on PASW-Windows (release 18.0; PASW, Chicago, IL, USA). All baseline data are expressed as mean (standard deviation (SD)). We used an analysis of covariance (ANCOVA) for the primary and secondary endpoints. The ANCOVA model included the outcome measure at baseline as the covariate and the repeat outcome measure (at eight or 26 weeks) and treatment allocation as the fixed factors. The data are presented as mean (95% confidence interval). The level of significance was set at a *p*-value of <0.05.

### Sample size

Due to the paucity of data on exercise training in dust-related ILD and no data on exercise training in people with pleural disease, the sample size was based on outcomes of exercise training in people with ILD (without IPF) [10]. Twenty-six participants were required to give the study a statistical power of 80% to detect a difference between groups in 6MWD following exercise training of 44 ± 34 m with a 5% significance level and allowing for 25% loss to follow-up.

## Results

### Participants

Forty-four male participants were assessed with 35 included in the study. Of these 35 participants, 24 (69%) had asbestos related pleural disease (ARPD), five (14%) had asbestosis, three (9%) had silicosis, two (6%) had combined ARPD and asbestosis, and one (3%) had mixed-dust pneumoconiosis. Thus the majority of participants had pleural not parenchymal lung disease. Only two participants had any evidence of COPD on high resolution computed tomography (HRCT) scan. The reasons for non-inclusion of nine participants were pain affecting exercise performance (two), neurological impairment (two), unstable cardiovascular responses during exercise testing (two), parietal pleural plaques only (one), peripheral vascular disease affecting exercise performance (one) and an inability to understand English (one). Baseline characteristics of the 35 participants and the diagnoses of study participants are shown in Table 1. There were no differences between the groups at baseline.

**Figure 1:**
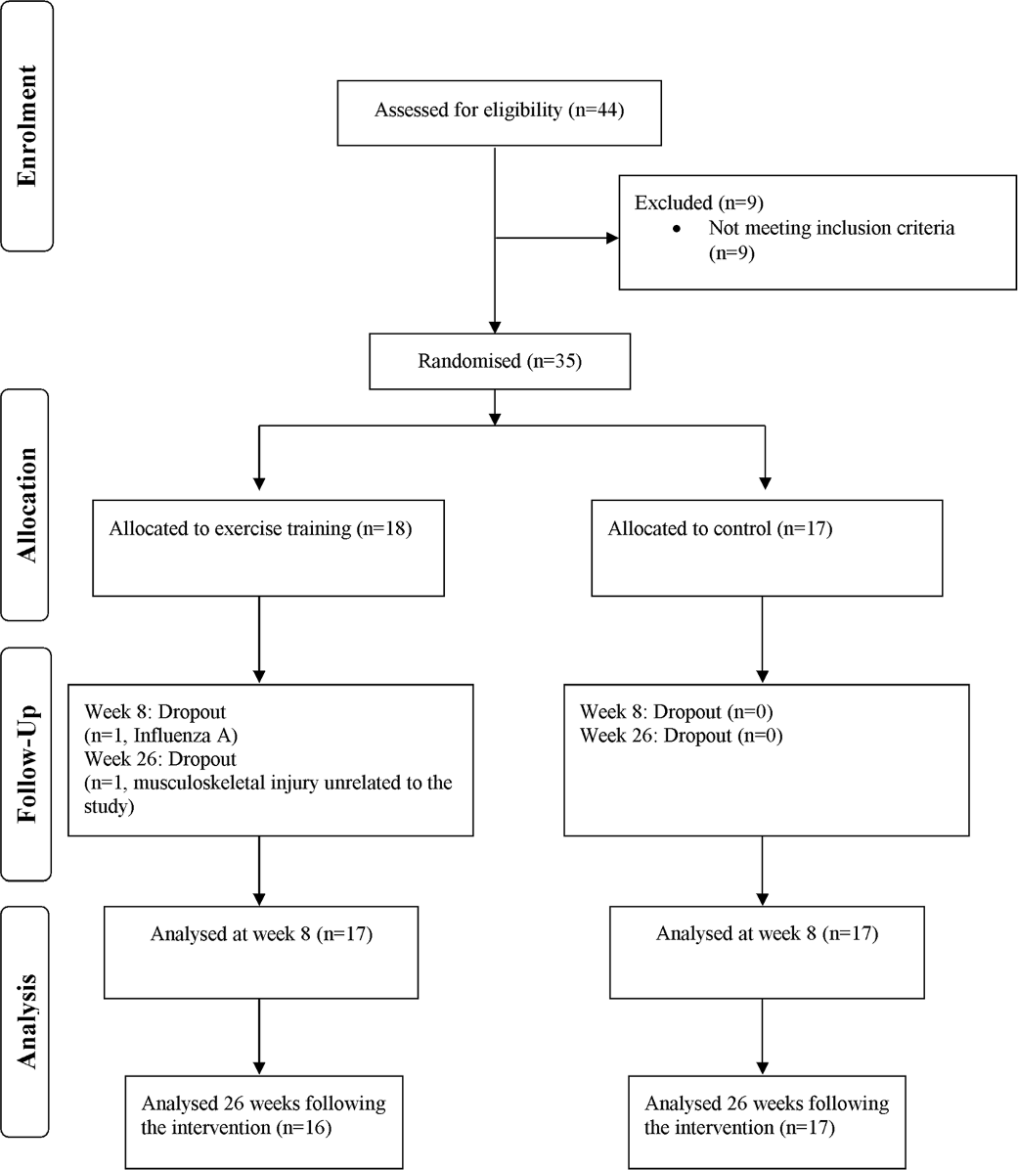
**Participant flow.**

At eight weeks, 34 participants completed the 6MWT and the HRQoL questionnaires, 33 completed the ICT and the ECT and 31 completed the physical activity measurements. At 26 weeks, 31 participants completed the 6MWT, 33 completed the HRQoL questionnaires and 30 completed the physical activity measurements. The flow of participants is presented in Figure 1. There was no significant change in FVC during the study (p = 0.823). No adverse events were reported during the exercise training.

**Table 1:** **Baseline characteristics, disease type, exercise capacity and health-related quality of life of the study participants**

	Exercise training group	Control group
	n =18	n =17
	Mean (SD)	Mean (SD)
Age, yr	70 (7)	72 (6)
Height, cm	170 (5)	175 (6)
Weight, kg	80 (13)	85 (11)
BMI, kg/m^2^	27 (4)	28 (3)
ARPD	11	13
Asbestosis	3	2
Asbestosis + ARPD	1	1
Silicosis	2	1
Mixed-dust pneumoconiosis	1	0
FVC, L	3.4 (0.9)	3.5 (0.8)
FVC, % pred	86 (23)	86 (18)
FEV_1_, L	2.1 (0.6)	2.4 (0.6)
FEV_1_, % pred	75 (23)	81 (16)
FEV_1_/FVC, %	64 (11)	69 (7)
FEV_1_/FVC, % pred	88 (16)	95 (10)
TLC, % pred	83 (20)	79 (15)
FRC, % pred	82 (28)	77 (15)
RV, % pred	77 (34)	72 (19)
D_L_CO, % pred	54 (15)	57 (13)
KCO, % pred	77 (22)	82 (19)
Smoking history, pack year	18 (18)	16 (25)
6MWD, m	474 (77)	469 (81)
SGRQ Total	30 (15)	29 (19)
SGRQ Activity	42 (21)	42 (28)
SGRQ Symptoms	38 (19)	32 (20)
SGRQ Impacts	20 (14)	20 (17)
CRQ Total	105 (13)	100 (13)
CRQ Dyspnoea	17 (7)	20 (6)
CRQ Fatigue	20 (5)	21 (3)
CRQ Emotional Function	40 (7)	40 (6)
CRQ Mastery	23 (4)	23 (4)
Average daily steps	7897 (2557)	8347 (3981)
Average daily EE, cal	2373 (349)	2660 (521)
Average daily METs	1.3 (0.1)	1.3 (0.2)

n = number; SD = standard deviation; yr = year; cm = centimetre; kg = kilogram; BMI = body mass index; m = metre; ARPD = asbestos related pleural disease; FVC = forced vital capacity; L = litre; % pred = percentage of predicted value; FEV_1_ = forced expiratory volume in one second; TLC = total lung capacity; FRC = functional residual capacity; RV = residual volume; D_L_CO = diffusing capacity for carbon monoxide; KCO = carbon monoxide transfer coefficient; 6MWD = six-minute walk distance; SGRQ = St George’s Respiratory Questionnaire; CRQ = Chronic Respiratory Disease Questionnaire; EE = energy expenditure; cal = calorie; MET = metabolic equivalent.

**Figure 2:**
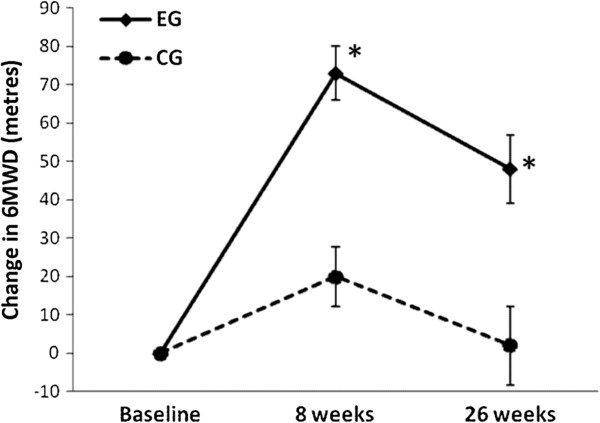
**Change in six-minute walk distance (6MWD).** Data are means and standard errors. *p < 0.05, exercise training group (EG) versus control group (CG).

Participants in the EG attended a mean (SD) of 20 (5) exercise training sessions. Fifteen participants were adherent with the exercise training sessions and three participants were non-adherent, attending three, nine and 17 training sessions respectively. The effects of exercise training on 6MWD at eight weeks are shown in Table 2 and Figure 2 and at 26 weeks are shown in Table 3 and Figure 2. The 6MWD was significantly greater in the EG compared to the CG at eight weeks, mean difference 53 m (95% CI 32 to 74). At 26 weeks the mean (SD) 6MWD for the EG was 525 (81) metres and for the CG was 466 (101) metres. There was a between group difference in the change in 6MWD from baseline to 26 weeks of 45 m (95% CI 17 to 73) (p = 0.003) which was significant in favour of the EG (Figure 2).

**Table 2:** **Mean (SD) of groups, mean (SD) difference within groups, mean (95% CI) difference between groups for exercise capacity immediately following intervention**

	Group				Difference within group		Difference between groups	
	Week 0		Week 8		Week 8 minus week 0		Week 8 minus week 0	ANCOVA
	Mean (SD)		Mean (SD)		Mean (SD)		Mean difference (95% CI)	p Value
**6MWT**	**EG**	**CG**	**EG**	**CG**	**EG**	**CG**		
	**n = 18**	**n = 17**	**n = 17**	**n = 17**	**n = 17**	**n = 17**		
6MWD, m	475 (79)	469 (81)	548 (72)	489 (83)	73 (29)	20 (32)	53 (32 to 74)	<0.0001*
**ICT**	**EG**	**CG**	**EG**	**CG**	**EG**	**CG**		
	**n = 18**	**n = 17**	**n = 16**	**n = 17**	**n = 16**	**n = 17**		
Peak work rate, W	108 (41)	110 (33)	116 (43)	107 (30)	8 (7)	-4 (12)	12 (5 to 19)	0.002*
VO_2_peak, L/min	1.6 (0.5)	1.6 (0.4)	1.6 (0.5)	1.6 (0.4)	0.1 (0.1)	0.1 (0.3)	-0.01 (-0.16, 0.14)	0.882
V_E_peak, L/min	56 (21)	55 (12)	59 (24)	55 (13)	3 (7)	0.3 (11)	2.84 (-4.13, 9.80)	0.412
HR at peak work rate, bpm	121 (22)	123 (13)	120 (23)	126 (12)	-0.5 (8)	3 (11)	-4 (-11, 3)	0.238
V_E_ at isowork, L/min	56 (21)	52 (12)	55 (24)	53 (13)	-0.6 (5)	1.3 (8)	-2 (-7, 3)	0.411
HR at isowork, bpm	121 (22)	119 (11)	117 (21)	122 (13)	-4 (9)	3 (8)	-7 (-13, -0.3)	0.040*
Borg dyspnoea at isowork	4 (2)	3(2)	4(2)	4(2)	0 (2)	1 (2)	-0.5 (-1.6, 0.6)	0.366
RPE at isowork	4 (3)	4 (2)	4 (2)	5 (2)	-0.4 (2)	0.2 (2)	-0.5 (-1.6, 0.5)	0.315
**ECT**	**EG**	**CG**	**EG**	**CG**	**EG**	**CG**		
	**n = 18**	**n = 17**	**n = 16**	**n = 17**	**n = 16**	**n = 17**		
Endurance cycle time, seconds	319 (130)	311 (80)	534 (268)	308 (113)	216 (254)	-3 (113)	222 (84, 360)	0.003*
V_E_ at isotime, L/min	51 (15)	53 (13)	50 (15)	52 (12)	-0.1 (5)	-1 (10)	0.6 (-5, 6)	0.824
Borg dyspnoea at isotime	5 (2)	3 (2)	3 (2)	4 (2)	-1 (2)	1 (2)	-1.2 (-2.3, -0.04)	0.043*
Borg dyspnoea at test end	5 (3)	4 (2)	5 (2)	5 (2)	-0.2 (1)	1 (2)	-1.1 (-2.3, 0.1)	0.076
RPE at isotime	5 (3)	5 (2)	4 (3)	5 (2)	-1 (2)	-0.3 (2)	-0.9 (-2.2, 0.3)	0.148

SD = standard deviation; CI = confidence interval; ANCOVA = analysis of covariance; 6MWT = six minute walk test; EG = exercise training group; CG = control group; n = number; 6MWD = six minute walk distance; m = metre; ICT = incremental cycle test; W = watts; VO_2_peak = peak oxygen uptake; L/min = litres per minute; V_E_peak = minute ventilation at peak work rate; HR = heart rate; bpm = beats per minute; V_E_ = minute ventilation; RPE = rate of perceived exertion; ECT = endurance cycle test; *p <0.05.

**Table 3:** **Differences between the exercise training and control groups immediately following intervention and 26 weeks following intervention for exercise capacity, health-related quality of life and physical activity**

Outcome	Week 8 minus week 0	ANCOVA	Week 26 minus week 0	ANCOVA
	Mean difference	p Value	Mean difference	p Value
	(95% CI)		(95% CI)	
**Exercise capacity**	**n = 34**		**n = 31**	
6MWD, m	53 (32, 74)	<0.001*	45 (17, 73)	0.003*
**HRQoL**	**n = 34**		**n = 33**	
SGRQ Total	-7 (-13, -1)	0.033*	-4 (-12, 4)	0.293
SGRQ Symptoms	-15 (-27, -2)	0.021*	-4 (-18, 9)	0.530
SGRQ Activity	-5 (-13, 3)	0.209	-4 (-15, 8)	0.505
SGRQ Impacts	-5 (-12, 2)	0.183	-4 (-12, 5)	0.383
CRQ Total	6.4 (2.1, 10.7)	0.005*	13.1 (5.2 to 20.9)	0.002*
CRQ Dyspnoea	2.2 (0.6, 3.7)	0.007*	3.4 (1.5, 5.4)	0.001*
CRQ Fatigue	1.3 (-0.02, 2.6)	0.053	2.9 (0.6, 5.2)	0.015*
CRQ Emotional Function	2.4 (0.7, 4.2)	0.008*	5.5 (2.0, 8.9)	0.003*
CRQ Mastery	1.4 (0.1, 2.7)	0.036*	2.7 (1.0, 4.3)	0.002*
**Physical activity**	**n = 31**		**n = 30**	
Average daily steps	1514 (15, 3012)	0.048*	1206 (-156, 2568)	0.08
Average daily EE, cal	86 (-57, 230)	0.227	158 (-8, 323)	0.062
Average daily METs	0.08 (0.01, 0.15)	0.038*	0.11 (0.03, 0.20)	0.012*

CI = confidence interval; ANCOVA = analysis of covariance; n = number; 6MWD = six-minute walk distance; m = metre; HRQoL = health-related quality of life; SGRQ = St George’s Respiratory Questionnaire; CRQ = Chronic Respiratory Disease Questionnaire; EE = energy expenditure; cal = calorie; MET = metabolic equivalent.

*p <0.05.

At eight weeks, peak work rate and endurance cycle time were significantly greater in the EG than the CG (Table 2). In addition, at eight weeks, heart rate at isowork rate on the ICT was significantly lower in the EG than the CG and Borg dyspnoea score at isotime on the ECT was significantly lower in the EG than the CG (Table 2).

HRQoL significantly improved in the EG compared to the CG at eight weeks (Table 3). The CRQ total score and domain scores for dyspnoea, emotional function and mastery and the SGRQ total and symptoms scores demonstrated significantly improved scores in the EG compared to the CG at eight weeks. At 26 weeks, the difference between groups in the CRQ total score and CRQ domains were maintained. However, there were no differences between groups in the SGRQ domains at 26 weeks (Table 3).

There were small statistically significant improvements in measures of physical activity (average daily steps and average daily METs) at eight weeks in the EG compared to the CG (Table 3). Although the improvement in average daily METs was maintained at 26 weeks in the EG, the difference between groups was very small, 0.11 METs (95% CI 0.03 to 2.0). At 26 weeks there was no significant difference in average daily steps between the EG and the CG.

## Discussion

This is the first randomised controlled trial to examine the effects of exercise training on exercise capacity and HRQoL in people with dust-related pleural and interstitial respiratory diseases. This study demonstrated improvements in exercise capacity and HRQoL immediately following an exercise training programme compared to a control group of usual care, with improvements in the exercise training group sustained at 26 weeks following intervention.

Exercise training improved functional exercise capacity measured by an increase of 53 metres in 6MWD in this cohort of people with dust-related pleural and interstitial respiratory diseases. This magnitude of change was larger than the 20 to 35 metre improvements reported in people with ILD [10, 11] and similar to the 48 metre improvement after exercise training in people with COPD [28]. In our study, the improvement in 6MWD was maintained 26 weeks after the completion of supervised exercise training without ongoing supervised exercise. This finding differs from the study by Holland et al where improvements in 6MWD were not maintained at 26 weeks in people with ILD, predominantly IPF [10]. One possible explanation is that our cohort included participants with mild to moderate dust-related pleural and interstitial respiratory diseases rather than more severe ILD. Our participants also had relatively stable disease over the study period, as evident by the stability of the FVC over 26 weeks, compared to the significant decline in FVC over a similar period in the study by Holland et al, indicating the progressive and severe nature of ILD in their cohort.

Exercise training also resulted in improvements in peak and endurance exercise capacity. Compared to the CG, the EG achieved a mean improvement of 12 watts in peak exercise capacity and a significant reduction in heart rate of 7 beats/minute at isowork rate. In the absence of changes to ventilatory parameters, these findings indicate that cardiovascular and peripheral muscle adaptations consistent with known training effects occurred. Participants in the EG also improved their endurance exercise capacity with a mean improvement of 222 seconds in endurance cycle time following exercise training and a reduction of perceived breathlessness at endurance isotime, which has not been previously reported in people with ILD. Therefore, exercise training in people with dust-related pleural and interstitial respiratory diseases yielded a similar improvement in endurance exercise capacity as those with COPD who demonstrated improvements in endurance cycle time of 198 (352) seconds following an exercise training programme [29].

The minimal important difference for improvements in exercise capacity in people with dust-related pleural and interstitial respiratory diseases has not been determined. However, the improvement in 6MWD in the EG in our study was considerably greater than the reported MID of 35 metres for the 6MWT in people with COPD [30] and the MID ranging from 29-34 metres in people with diffuse parenchymal lung disease [31]. The improvements in endurance cycle time and peak work rate in people with dust-related pleural and interstitial respiratory diseases were also greater than the MID ranging from 100-200 seconds [29] and 3-5 watts [32] respectively in people with COPD. Therefore, the use of a similar exercise training regimen for people with dust-related pleural and interstitial respiratory diseases, as has been previously used in COPD [17], resulted in improvements in exercise capacity that exceeded the MID established for people with COPD or other ILD.

Important improvements in HRQoL were achieved in people with dust-related pleural and interstitial respiratory diseases immediately following exercise training. The improvement in SGRQ total score was greater than the clinically important reduction of 4 points for people with COPD [33]. Despite statistically significant improvements in the CRQ total score and domains of dyspnoea and emotional function, these improvements did not reach the MID established for COPD. Twenty-six weeks following the intervention the significant difference between groups was maintained and interestingly the values of the EG group increased further and reached the MID in all domains of the CRQ. The improvements in HRQoL occurred with exercise training, in the absence of an education component, as has previously been shown in COPD [34]. This study has also demonstrated that the SGRQ and CRQ, which are disease specific questionnaires originally designed to assess HRQoL in people with COPD, can also be used to detect changes in HRQoL in people with dust-related pleural and interstitial respiratory diseases.

This is the first study to evaluate the effects of exercise training on levels of daily physical activity in people with dust-related pleural and interstitial respiratory diseases. In epidemiological studies, higher levels of physical activity have been associated with health benefits in people with COPD [14, 15] but this has not been evaluated in ILD or pleural respiratory diseases. Our study demonstrated small but significant improvements in physical activity after exercise training compared to a control group of usual care, both in the short-term (eight weeks) and the longer term (26 weeks). A recent meta-analysis demonstrated a very small increase in physical activity after exercise training in people with COPD [35]. Although the improvement in average daily METs in our study was maintained following exercise training in the longer term, the small magnitude of improvement questions the clinical relevance of this finding. More research is needed to identify how best to improve activity levels in this patient population and whether improvements in physical activity are associated with improved health outcomes.

The development of dust-related pleural and interstitial respiratory diseases is characterized by a long latency period from exposure to dust to development of disease [3]. Consequently, the mean age of participants was 71 years. With increasing age, the FEV_1_/FVC ratio decreases. While the mean FEV_1_/FVC ratio in our participants was less than 0.7, FEV_1_/FVC ratio as a percent of predicted was within the normal range for people of this age [21] and did not reflect the presence of COPD. The HRCT findings further confirmed that only two participants had any evidence of COPD. Thus the findings of this study are specifically related to asbestos-related and other dust-related respiratory disease and are not confounded by co-existing COPD.

The original sample size calculation was based on outcomes of exercise training in people with ILD. As the study progressed, two distinct participant groups became evident, participants with dust-related pleural disease and participants with dust-related interstitial lung disease. To account for this heterogeneity, more participants were recruited than originally calculated.

There are several limitations to this study. Incremental and endurance cycle ergometry tests were not repeated at 26 weeks thus it is unknown whether improvements in peak and endurance exercise capacity shown at eight weeks were maintained in the longer term. In addition, participants did not complete resistance training as part of the exercise training therefore the effects of resistance training in this population remain unclear. While recruitment was open to males and females, only males volunteered to participate in the study, so the findings cannot be extrapolated to females with dust-related respiratory diseases. Since our study population did not include people with dust-related pleural and interstitial respiratory diseases who were on long term oxygen therapy and as our participants did not have severe disease, the findings of our study cannot be extrapolated to such sub-groups. Finally, there was some heterogeneity in the diagnoses of study participants which limit the generalisation of the findings. More studies of each specific disease group are needed to address the paucity of literature on the specific effects of exercise training in people with dust-related pleural disease and dust-related interstitial lung disease. Such diseases usually have a slower progression than other ILDs and thus interventions such as exercise training that improve exercise capacity, quality of life and levels of daily physical activity may have an important role in the management of these diseases that previously have had few effective therapies.

## Conclusion

This randomised controlled trial demonstrated that exercise training improved short and long-term exercise capacity and health-related quality of life in people with dust-related pleural and interstitial respiratory diseases. Commonly, people with dust-related pleural and interstitial respiratory diseases have not been considered appropriate for referral to pulmonary rehabilitation programmes due to the lack of evidence of benefit. This study has provided compelling evidence for the benefits of exercise training in people with dust-related pleural and interstitial respiratory diseases and for the inclusion of this population in pulmonary rehabilitation programmes.

## Abbreviations

HRQoL: Health-related quality of life
ILD: Interstitial lung disease
IPF: Idiopathic pulmonary fibrosis
COPD: Chronic obstructive pulmonary disease
D_L_CO: Diffusing capacity for carbon monoxide
EG: Exercise training group
CG: Control group
FVC: Forced vital capacity
DDB: Dust Diseases Board
6MWT: Six-minute walk test
ICT: Incremental cycle test
RPE: Rate of perceived exertion
RPAH: Royal Prince Alfred Hospital
6MWD: Six-minute walk distance
CRQ: Chronic Respiratory Disease Questionnaire
SGRQ: St George’s Respiratory Questionnaire
ECT: Endurance cycle test
VO_2_: Oxygen uptake
VCO_2_: Carbon dioxide output
SpO_2_: Oxygen saturation
MID: Minimal important difference
MET: Metabolic equivalent
SD: Standard deviation
ANCOVA: Analysis of covariance
ARPD: Asbestos related pleural disease
HRCT: High resolution computed tomography
CI: Confidence interval
FEV_1_: Forced expiratory volume in one second.
